# Supplementary material for: Covid-19 Transmission Trajectories–Monitoring the Pandemic in the Worldwide Context
Source: Viruses. 2020 Jul 20;12(7):777. doi: 10.3390/v12070777 (PMC7412525; doi:10.3390/v12070777)
Supplement: Supplementary file 1 [file viruses-12-00777-s001.pdf]

Supplementary Material

Covid-19 trajectories – Monitoring pandemic in the worldwide context

Henry Loeffler-Wirth [1]*, Maria Schmidt [1], Hans Binder [1]*

[1] IZBI, Interdisciplinary Centre for Bioinformatics, Universität Leipzig, Härtelstr. 16 – 18, 04107 Leipzig, Germany

* Authors to whom correspondence should be send: Wirth@izbi.uni-leipzig.de; Binder@izbi.uni-leipzig.de

Rate factors and trajectories

Time-dependent rate factors and reproduction numbers:

Covid-19 case numbers are provided as country-wise time series of the daily numbers of newly infected and removed (recovered plus died) cases (both in units of 1/day), $\Delta N(t)$ and $\Delta R(t)$, respectively. Let us define so-called rate factors of transmission and removal of infected cases and of their ratio as

Effective transmission rate factor: $c_e(t)= \Delta N(t)/ I(t)$,

Removal rate factor: $k(t)= \Delta R(t)/ I(t)$, (1)

Ratio (effective reproduction number) $R_e(t)= c_e(t)/ k(t)$,

where I(t) denotes the current number of infected cases. The meaning of the ratio as effective reproduction number can be obtained from the SIR model discussed in Appendix II (see also [43]). Rate factors and reproduction numbers were not calculated if daily case numbers (infected and/removed) are less than ten.

Biased case numbers:

Usually case numbers (infected, recovered, death) are biased due to different, country-dependent census criteria, e.g., for counting death cases (as related to Covid-19 or not) or infected ones (e.g. by considering only hospitalized and/or tested persons), due to different test frequencies and, probably, also using tests of different quality, e.g. regarding their specificity. Considering that usually only a (constant) part p of the newly infected cases (ΔN) are detected, then all other numbers scale accordingly, i.e.,

$\Delta N^*= p\, \Delta N$, $N^*= p\, N$, $I^*= p\, I$, $R^*= p\, R$ and $P^*= p\, P$,

where the scaled values are denoted by the asterisk, ‘*’, assigning the ‘visible’ cases and the ‘visible’ population (P*). ‘Proportional’ scaling don’t affect estimation of the rate factors,

$c_e= \Delta N^*/ I^* = \Delta N/ I$, $k= \Delta R^*/ I^*= \Delta R/ I$

In other words, rate factors (and consequently also their ratio, the effective reproduction numbers, R_e) represent relative numbers which remove country-specific biases in case of proportional scaling or, at least, decrease biasing effects if proportional scaling condition do not strongly hold. Hence, trajectory plots and derived numbers are largely independent of the case detection methods which are known to vary both from country to country as well as over time for a given country. Throughout the paper we understand case numbers as visible ones and we therefore omit '*'.

Trajectories:
With R(t), the number of removed cases, and N(t)= I(t) + R(t), the cumulative number of infected cases, one can rewrite Equation (1) as follows to obtain expressions for the trajectories used in this paper (where we omit the time as argument for sake of briefeness):

Log-log trajectory: y = 'vertical shift' + 'slope' *x

ΔN-vs-N trajectory (rise-fall trajectory): $\log \Delta N = \log(c_e - k) + \log N$

ΔN-vs-I trajectory: $\log \Delta N = \log c_e + \log I$. (2)

ΔR-vs-I trajectory: $\log \Delta R = \log k + \log I$

The y-vs-x trajectory plots provide an intuitive approach to describe the dynamics of epidemic by simple visual inspection of the graphs obtained. Linear graphs with a slope of unity are indicative for exponential changes of the respective case numbers and thus, virtually constant values of the respective rate factors. They become rate 'constants' in this situation determining the steepness of exponential changes. The vertical shift of the linear parts of the trajectories with respect to the diagonal line, y= x, relates to the values of the rate constants ($c_e - k$), c_e and k for the rise-fall, ΔN-vs-I and ΔR-vs-I trajectories, respectively. Reduction of the rate constants will result in a parallel, downwards shift of the respective linear trajectories, while increasing rate factors will shift the lines upwards. Non-linear, curved trajectories are indicative for temporal changes of the rate 'constants', which become variable rate 'factors' in these situations. Downwards curved trajectories are indicative for a decay of the respective rate factors and upwards curved trajectories are indicative for increasing rate factors. Lines with slopes deviating from unity indicate rather power law then exponential time dependencies [27].

Hence, the ΔN-vs-I (newly confirmed infections versus current infections) and ΔR-vs-I (actually removed cases versus current infections) trajectories visualize the behaviour of the transmission and removal rate factors, respectively, while the 'rise-fall' trajectory (new infections versus cumulative infections) reflects their difference. Its upwards shift refers to ($c_e - k > 0$) meaning that the epidemic will increase spreading, while its downwards shift, i.e., ($c_e - k < 0$), indicates its shrinkage. With ($c_e - k$) = k ($R_e - 1$) one finds for these situations $R_e > 1$ and $R_e < 1$, respectively, and consequently $R_e = 1$ for the epidemic threshold (ET) separating them. In summary, the trajectory-approach makes use of the transformation of case numbers into rate factors (Equation (1)) and their plot as described in Equation (2). Formally these plots do not require any epidemic model, however such models can support interpretation of the trajectories obtained.

SIR model

The Susceptible-Infected-Removed (SIR) model divides a population of size P into three categories, healthy and susceptible to the disease (S), infected (and thus infectious, I) and people who were removed (R) from infection due to recovery (healthy and not susceptible due to immunity) or death. The total population (including the deaths) is constant, P = S + I + R. As the disease progresses, susceptible individuals (S) after contacting infected individuals (I) are liable to get infected. Afterwards the infected (I) either die or recover, i.e. S → I → R, where notations are used as in chemical reaction kinetics. The arrows denote the transitions proceeding with rate constants c and k (cases per day), respectively. The time-derivatives of the numbers are given by:

Susceptible: $dS/dt = - c\, S / P\, I$

Infected: $dI/dt = (c\, S/P - k)\, I$ (3)

Removed: $dR/dt = k\, I$

Newly infected: $dN/dt=-dS/dt$ with $I_0= N(t=0)$

Here, c is the transmission rate constant and k the removal rate constant given in individuals per time step (our data use daily changes, i.e., dt = 1 day). The reciprocal rate constant $1/c_e$ defines consequently the average time spend before an infecteous person transmits (infects) a susceptible one. The early outbreak limit refers to early phases of epidemic if $S \approx P$. The solution of the equations (3) then provides an exponential growth function for infected individuals: $I(t) = I_0 \exp(+ (c - k)\, t)$, with the initial number of infected individuals I_0. Upon progression of the disease the number of susceptible individuals S(t) decays and the growth of I(t) gets retarded. The cumulative number of infected cases (N = P - S), represents the sum over the newly infected individuals per day.

By denoting all daily changes dX/dt as ΔX (X= N, S, I, R) one obtains estimates of the rate constants,

$c = (S/P)^{-1}\, \Delta N/ I$ and, $k= \Delta R/ I$. (4)

Comparison with Equation (1) relates the SIR rate constants to the rate factors,

$c_e(t)= c\, S(t)/P$ and $k(t)= k$. (5)

Hence, $c_e(t)$, the transmission rate factor is expected to decay with progressing epidemic due to the depletion of susceptible individuals, while the removal rate factor is a constant in the frame of the SIR model. In the early outbreak limit one gets $c_e(t=0)= c$, i.e. also the transmission rate factor becomes a constant. Note, that the transmission (and removal) times in the sense of the average days to transmit the infection to a person (remove a person from infected compartment after infection) is defined by the reciprocal values of the rate factors, i.e., $\tau_c = 1/ c_e$ ($\tau_k= 1/ k(t)$), respectively.

Theoretical rise-fall trajectories illustrate the effect of different SIR-model parameters (Figure S 1a).

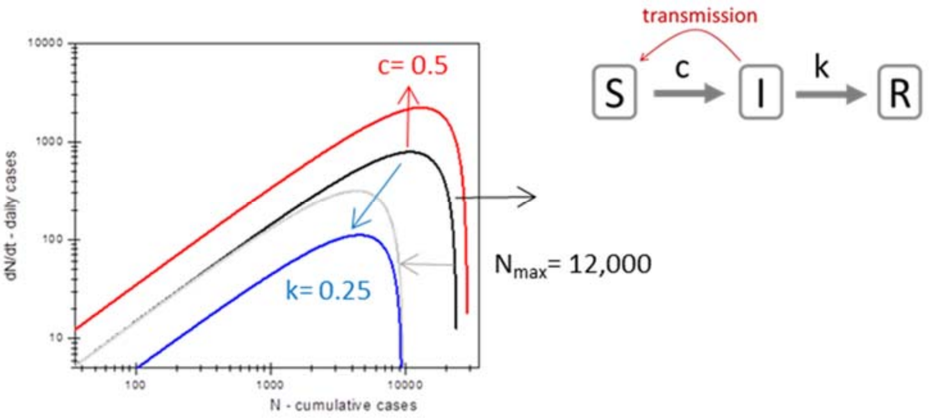

Figure S 1: Modeling ‘rise-fall’ trajectories: a) SIR model: Systematic variations of parameters assigns vertical parallel shifts of the rise-regime to the reduction of the transmission rate, c, and the onset of the fall-regime to k and/or Nmax.

SIR model curves: Equation (3) rewrites for the trajectories in Equation (2) into

$$\Delta N = c\,(1 - N/P)\,(N - R) \quad , \quad \text{with } R = -P/R_0 \ln(1 - N/P) \ \text{ and } \ N = I + R \quad . \qquad (6)$$

It enables calculating the number of newly infected individuals per day, ΔN, as a function of the cumulative number of infected individuals, N, using the transmission rate constant, c, the basic reproduction number, R_0= c/k (note that reproduction numbers have indices while the number of removed cases is simply denoted by ‘index-less’ R) and the population size, P, as parameters.

Variation of SIR-parameters (Figure S 1a): Variation of the model parameters indicates that the transmission rate, c, shifts the ‘rise-fall trajectory in vertical direction (red curve) while the maximum number of cases Nmax shifts the falling part horizontally (grey curve). Variation of the removal rate k affects both dimensions: Its increase effectively shifts the increasing part downwards and the falling part to the left. Accordingly, activation of NPI are expected to shift the trajectory downwards if they reduce c, the transmission rate constant.

__Epidemic threshold and reproduction numbers:__

The (time-dependent) transmission rate factor, $c_e(t)$, is an important measure for the spreading of an epidemic depending on the actual amount of susceptible individuals according to Equation (5). The condition $c_e(t)$ = k will held the number of infected persons constant (ΔI= 0). It defines the epidemic threshold (ET). The ratio R_e= $c_e(t)$/k estimates the apparent ‘effective’ reproduction number. It defines the number of susceptible persons getting infected by contact with one infected one [43]. The basic reproduction number refers to the early outbreak limit, i.e. R_0= R_e(t=0)= c/ k. The disease will invade a population for R_e> 1 and it will disappear for R_e< 1, respectively. This latter condition can be rewritten in the form S/P= (P − N_{max})/P < (1 − H) = 1/R_0, where N_{max} is the number of cumulative cases when epidemic stops. H= N_{max}/P = (1 − 1/R_0) is often called the herd immunity threshold estimating the fraction of immunized individuals meeting the ET condition. For Covid-19 typical values of R_0= 2.5 – 3 [12] one obtains H= 0.6 - 0.7. These simple relations assume random contacts followed by transmission in the population, which is an oversimplification because of spatial (e.g. between cities and countryside), social (e.g. intra- and extra-familial contacts and their age-dependent changes), and economical (e.g., working environments) heterogeneities in a population. A possible decrease of the herd immunity threshold in heterogeneous populations was recently discussed [44].

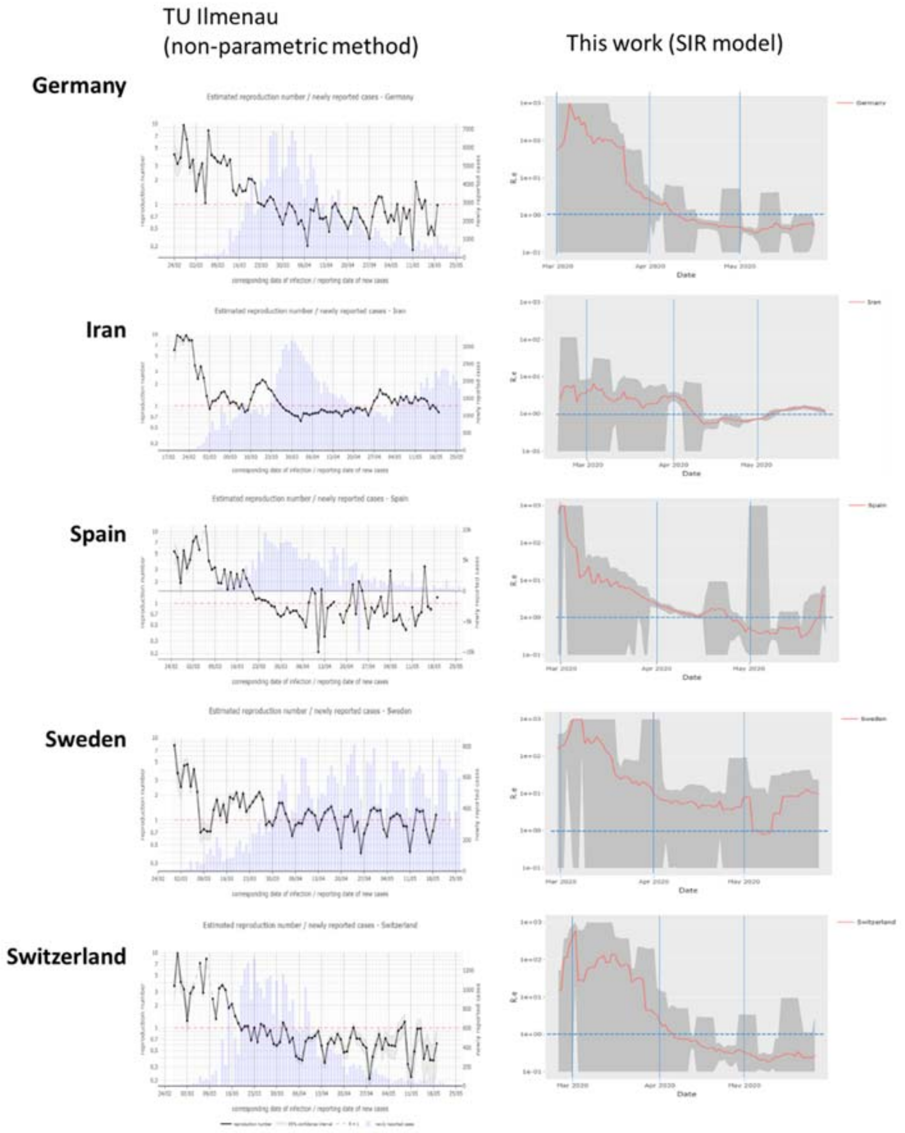

Figure S 2: Effective reproduction of selected countries as a function of time were compared between two methods. **Left part**: Plots of $R_e(t)$ were taken from https://stochastik-tu-ilmenau.github.io/COVID-19/all.html . They were calculated using the non-parametric method as proposed in [45]. The data were shift by 2 weeks to an earlier date to account for the time span between infection and recovery. **Right part:** $R_e(t)$ were calculated using Equation (1). The 7-days sliding window used for averaging largely removes the weekly oscillation seen in the daily data shown at the right.

<u>The effect of NIPs on the rate factors:</u>

NPIs (Non-Pharmaceutical Interventions) include measures such as lock down of community and economic activities, social distancing, face masks wearing and/or isolation of infected individuals. According to Equation (5) these measures are expected to affect c (e.g. social distancing, face masks, reduction of contacts) or S (isolation, stop traveling, heterogeneity of population with respect to transmission and mixing). In general, both values become affected by NPIs in a time-dependent fashion giving rise to

$$c_e(t) = m(t)\ c\ S(t)/P \qquad (6)$$

where m(t)< 1 is the 'measures' function describing the reduction of $c_e(t)$, where m(t) subsumes effects on c and S as well. As a simple example, let us illustrate NPIs as isolation of part of susceptible persons (e.g. by locking down their contacts) and/or putting part of infected cases into quarantine. In general, measures were expected to decay the rates factors in terms of time depending rates c(t) and k(t) in the SIR model as proposed also previously [46].

Using custom trajectories to estimate transmission and removal rate constants:

The custom trajectories visualize the transmission and removal rates by plotting the incremental infected and removal cases as a function of the current number of infections, ΔN-vs-I and ΔR-vs-I, respectively. Both types of trajectories show a turning point for selected Middle (Germany, Austria, Switzerland) and Western (France, Italy, Spain) countries referring to the maximum number of infected cases in the respective countries (Figure S 3a). The ΔN-vs-I trajectories turn in clockwise direction giving rise to a downwards shift of its returning part. This behaviour reflects a marked decay of the transmission rate seen directly in the $c_e(t)$ graphs obtained from the trajectories using Equation (1) (Figure S 3b). In contrast, the ΔR-vs-I trajectories of the Middle European group of countries turn in counter-clockwise direction reflecting the marked increase of the removal rate factor seen directly in the graphs of k(t). The Western European countries and US don't show this left turn of the trajectory due to considerably smaller values of the removal rate factors compared with that of the Middle European countries. The reason of this difference is not clear. The smaller values mean slower recovery of Covid-19 cases in Spain, France, Italy and US compared with Germany, Austria and Switzerland. Note also that the effective reproduction numbers decay faster in the Middle European countries compared with Western Europe and US (Figure S 3b). In summary, custom trajectories ΔN-vs-I and ΔR-vs-I directly evaluate transmission and removal of epidemic in terms of the respective rate factors and their ratio, estimating the effective reproduction number.

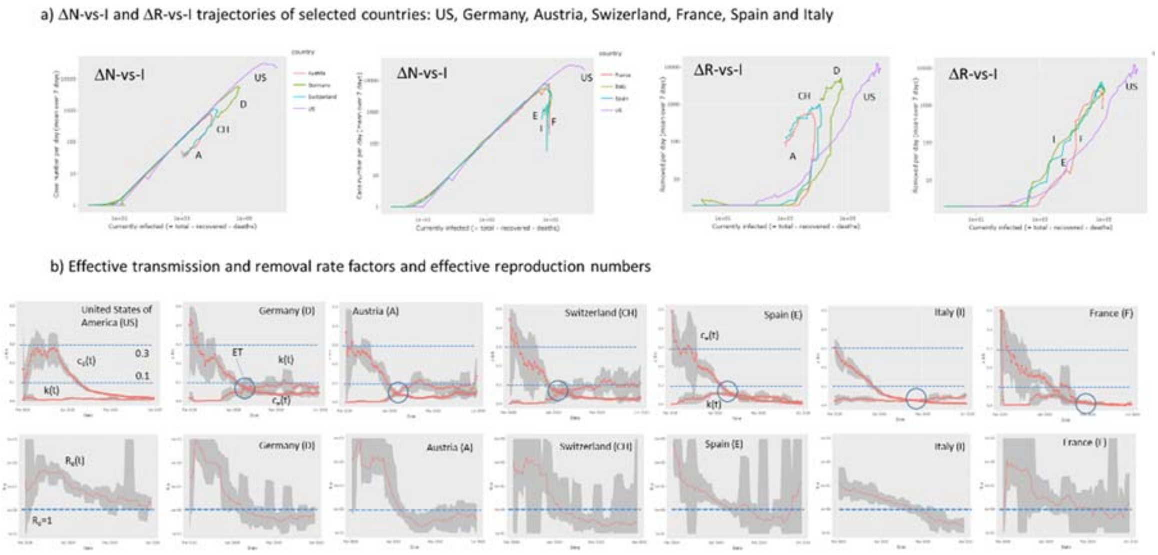

Figure S 3: Estimation of the transmission and removal rate constants of selected countries using custom trajectories: a) ΔN-vs-I and ΔR-vs-I trajectories of Germany, Austria and Switzerland (Middle European group) and of Italy, France and Spain (Western European group) and of USA for comparison. b) The relative increments of the trajectories estimate the effective rate factor of transmission and removal, respectively (Eq. (1)), and the effective reproduction rate. Except for US epidemic is stopped at the epidemic threshold (ET) if the course of $c_e(t)$ crosses that of k(t) (circle).

Supplementary Figures

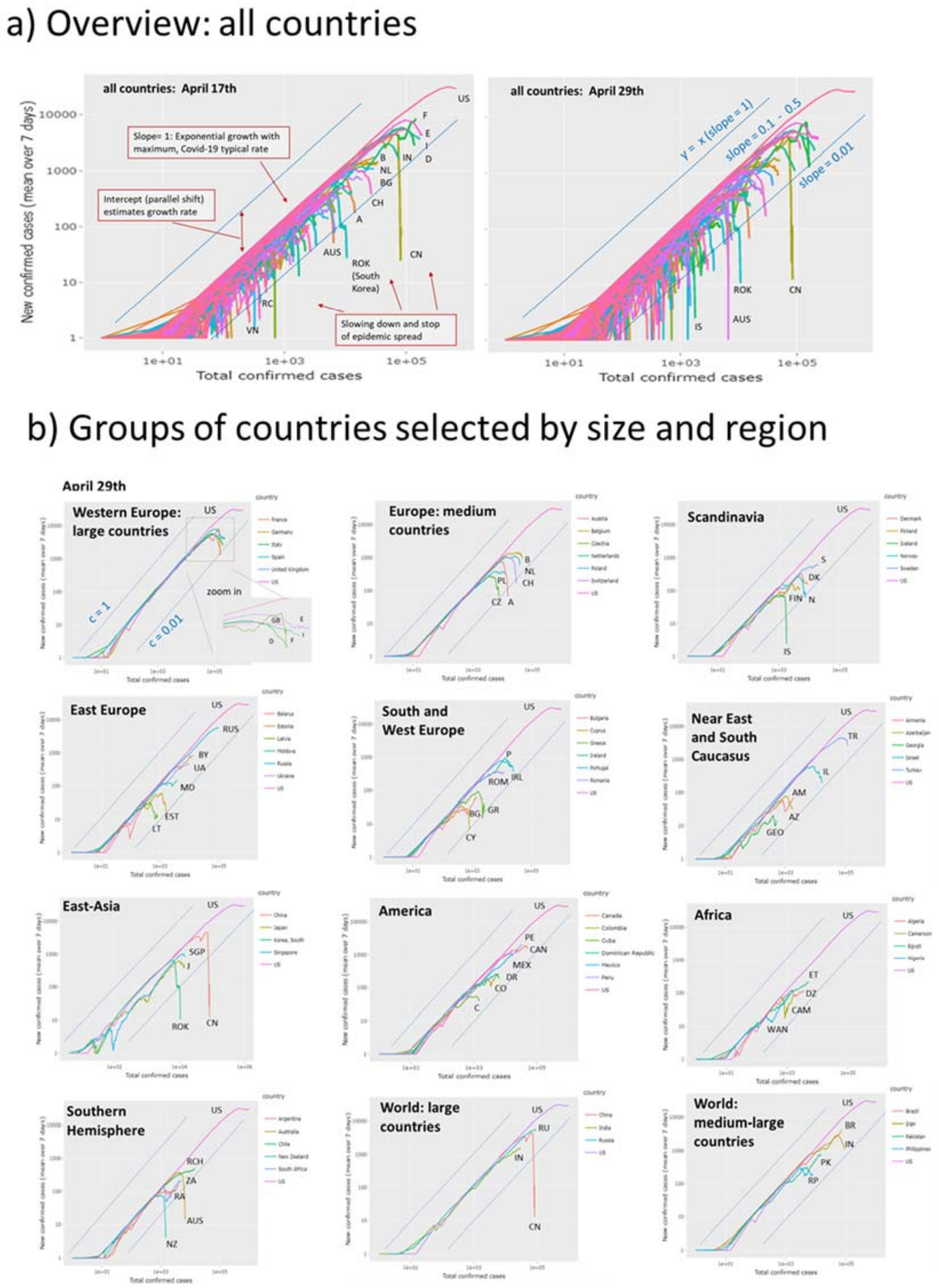

Figure S 4: Rise-fall trajectories as shown in Figure 4 for April 19th 2020 for comparison.

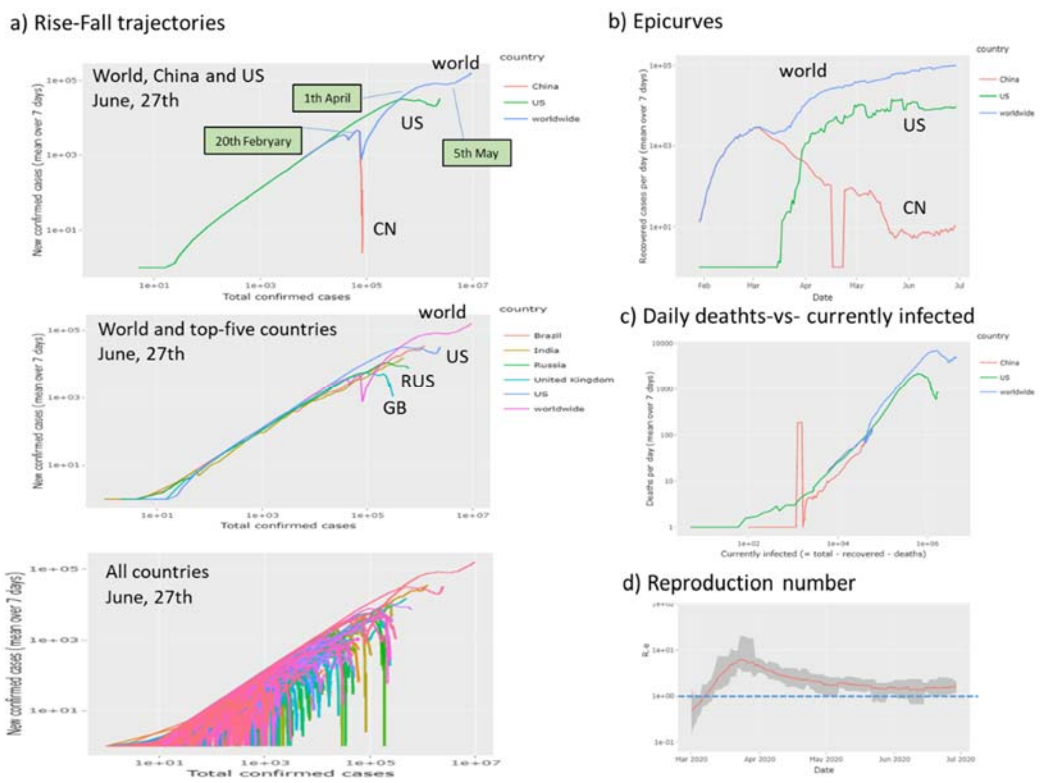

Figure S 5: The worldwide situation of Covid-19 a can be followed using the cumulative cases of all countries (select 'worldwide'): a) The rise-fall trajectory of the 'world' dropped around February 20th due to the stopp of epidemic in China. Presently pandemic is still growing exponentially after a temporal slowing down around May 5th (plot above). Three of the top-five countries with most Covid-19 cases show growing trends (plot in the middle), while the majority of countries shows a decaying trend (plot below). B) The time courses (epicurves) refer to the plot in part a. c) The increase of daily deaths is stopped in US and worldwide. d) The worldwide reproduction number is still larger than one (horizontal dashed line).

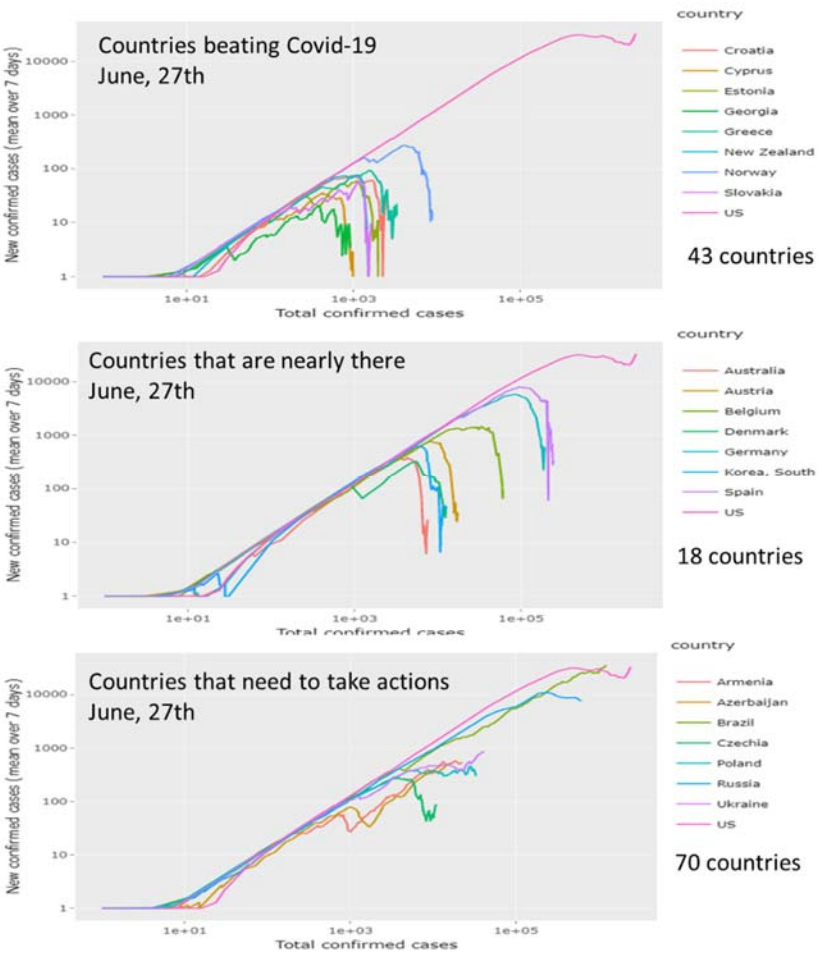

Figure S 6: Rise-fall trajectories of countries selected from three categories of countries regarding their present status of 'fighting against Covid-19' taken from https://www.endcoronavirus.org/countries). The respective status can be deduced by simple inspection of the trajectories. 'Beating Covid-19' associates with the steep drop of the falling part to only a few (usually a few dozen or less) new cases per day. Countries 'nearly there' show a clear drop where the number of new cases dropped by about two orders of magnitude compared with its maximum value. Countries 'need to take actions' if their trajectories are still (or again) in their rising part.

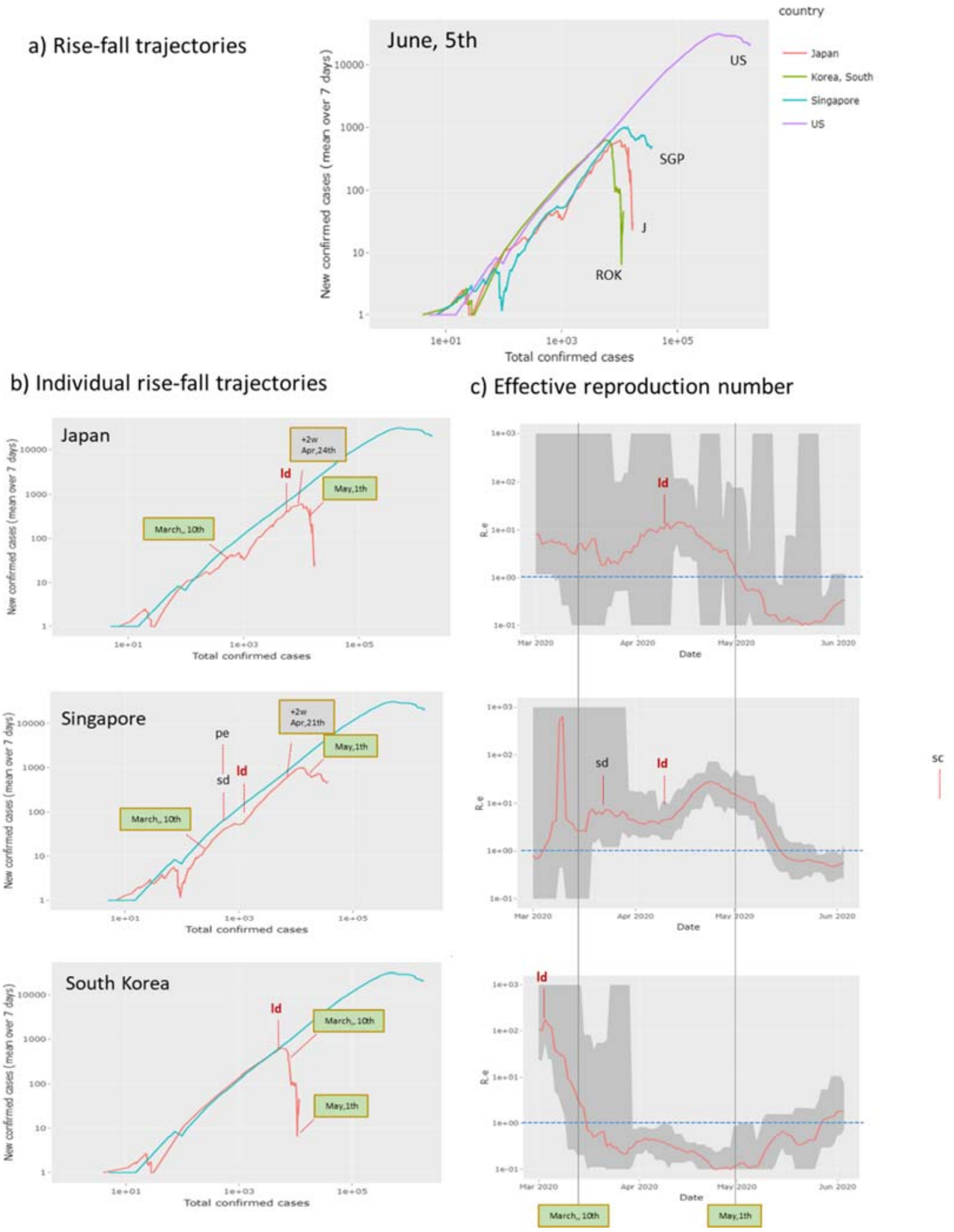

Figure S 7: Dating of NIPs in selected Asian countries along the rise-fall trajectories (a and b) and time courses of effective reproduction numbers (c). Sharp lockdown-like measures were applied relatively early in South Korea followed by the strong decay of new cases and also of R_e.Note that meanwhile (beginning of June) local outbreak custers give rise to the increase of R_e. Japan and Singapore show the slowing down of still growing transmission during March as indicated by the downshift of the rising part of the trajectory. In Japan the trajectory and R_e-course turn down after intensification of NIPs around April 10th, where similar to European countries, the data indicate stop of epidemic about two weeks after the measures were taken. In Singapore the down-turns of the curves were delayed after the lock-down compared with the other countries shown. Note that the character of NPIs and particularly, the 'lockdown' differ considerably between the countries and need more detailed analysis to establish causal relationships and to relate particular measures to the respective effect.

References

See main paper

and supplementary references

44. Ma, J., Estimating epidemic exponential growth rate and basic reproduction number. *Infectious Disease Modelling* **2020,** 5, 129-141.
45. Britton, T.; Trapman, P.; Ball, F. G., The disease-induced herd immunity level for Covid-19 is substantially lower than the classical herd immunity level. *medRxiv* **2020**, 2020.05.06.20093336.
46. Fraser, C., Estimating Individual and Household Reproduction Numbers in an Emerging Epidemic. *PLOS ONE* **2007,** 2, (8), e758.
47. Chen, Y.-C.; Lu, P.-E.; Chang, C.-S.; Liu, T.-H., Time-dependent SIR model for COVID-19 with Undetectable Infected Persons. *arXiv.org* **2020,** arXiv:2003.00122.
